# Assessment of motivating factors associated with the initiation and completion of treatment for chronic hepatitis C virus (HCV) infection

**DOI:** 10.1186/1471-2334-13-234

**Published:** 2013-05-23

**Authors:** Lauren Fusfeld, Jyoti Aggarwal, Carly Dougher, Montserrat Vera-Llonch, Stephen Bubb, Mrudula Donepudi, Thomas F Goss

**Affiliations:** 1 Boston Healthcare Associates, Inc., Boston, MA, USA; 2 Vertex Pharmaceuticals Incorporated, Cambridge, MA, USA

**Keywords:** Hepatitis C, Decision-making, Adherence, Compliance, Motivation, Treatment initiation, Drug therapy

## Abstract

**Background:**

Infection with hepatitis C virus (HCV) is associated with high morbidity and increased mortality but many patients avoid initiation of treatment or report challenges with treatment completion. The study objective was to identify motivators and barriers for treatment initiation and completion in a community sample of HCV-infected patients in the United States.

**Methods:**

Survey methods were employed to identify factors reported by patients as important in their decision to start or complete HCV treatment. Study participants included 120 HCV-infected individuals: 30 had previously completed treatment with pegylated interferon/ribavirin (PR), 30 had discontinued PR, 30 were treated with PR at the time of the survey, and 30 were treatment‒naïve. Telephone interviews occurred between May and August of 2011 and employed a standardized guide. Participants assigned factors a rating from 1 (not at all important) to 5 (extremely important). Trained researchers coded and analyzed interview transcripts.

**Results:**

Of 33 factors, expected health problems from not treating HCV infection was reported as most encouraging for treatment initiation and completion, while treatment side effects was most discouraging. Sixty-nine percent of participants reported that the ability to obtain information during treatment on the likelihood of treatment success (i.e., results of viral load testing) would motivate them to initiate therapy. Median preferred timing for learning about test results was 5 weeks (range: 1–23 weeks).

**Conclusion:**

Understanding challenges and expectations from patients is important in identifying opportunities for education to optimize patient adherence to their HCV treatment regimen.

## Background

Hepatitis C is a liver disease resulting from infection with the hepatitis C virus (HCV); the estimated disease prevalence in the United States is 5 million [1]. Acute hepatitis C occurs within the first six months after exposure to the HCV, and 75% to 85% of subjects become chronically infected. Most patients do not exhibit any symptoms (e.g., fatigue, fever, loss of appetite, nausea, vomiting, abdominal pain, joint pain, and jaundice) [2,3]. About 65% of all HCV-infected patients experience chronic liver damage, which can eventually lead to serious complications, including death [3,4].

Until 2011, the standard of care for patients with genotype 1 chronic HCV infection was a dual combination regimen of pegylated interferon and ribavirin (PR) [5]. Pegylated interferon is injected once weekly and ribavirin is taken orally twice a day with food. In this patient population, the typical duration of therapy was 48 weeks [5-7] and resulted in a sustained virologic response (SVR) in 40% to 50% of patients [5]. Treatment with PR is associated with significant side effects, in particular anemia, fatigue, and depressive symptoms.

Two direct-acting antiviral agents (DAA) – telaprevir and boceprevir – were approved for use in the United States and other countries in 2011 for the treatment of adults with genotype 1 HCV with compensated liver disease. Treatment with a DAA in combination with PR has improved SVR rates for treatment naïve patients with genotype 1 HCV infection to around 60% to 75% [5]. Furthermore, these new treatment regimens have allowed for shorter duration of therapy (i.e., 24 weeks) in eligible patients [5]. Increased treatment options with greater clinical efficacy than PR alone have renewed interest in understanding patient behavior and decision-making associated with HCV treatment initiation and completion. As DAA-based regimens include interferon and ribavirin, completing treatment remains challenging for many patients.

Several approaches to understanding the physical, demographic, emotional, and social factors that might motivate patients to start HCV therapy have been described in the literature, including those that focus on the structure of the decision making (e.g., risk-benefit review) [8]. Reasons associated with the decision to initiate treatment include the following: expectations of treatment effectiveness, threat of disease progression, the availability of care, lack of alcohol dependence, and readiness to stop using drugs [9,10]. Conversely, studies have shown that common deterrents to the initiation of HCV treatment include lack of disease symptoms, anticipated side effects from treatment, and the presence of comorbid conditions [11]. To date, many studies have focused on specific high-risk patient populations (e.g., illicit drug users) and other patient subgroups (e.g., patients without human immunodeficiency virus and treatment-naïve patients) [9-13].

An additional component of successful treatment for HCV is the ability to remain motivated throughout the course of therapy. Several studies have shown that patients reported challenges in adhering to a PR treatment regimen because of side effects, worsening fatigue, and declines in health-related quality of life [10,14]. Decreased treatment adherence and treatment discontinuation are associated with a reduced probability of virologic response [15,16].

The objective of this investigation was to identify factors reported by HCV-infected individuals in the United States as important in their decision to start and complete the prescribed HCV treatment. Study findings should inform the development of interventions in support of HCV treatment initiation and maintenance as new treatment options emerge for this patient population.

## Methods

Study procedures were reviewed by New England Institutional Review Board (NEIRB), an independent Institutional Review Board (IRB), and the study was determined to be exempt from review. All participants were informed about the study’s purpose and methods. Individuals provided verbal consent for participation; those who completed all study related activities received a stipend of $75.

### Study population

Seven hundred and seventy individuals were screened from a United States community convenience sample, of whom 123 HCV-infected patients were eligible for the study and 120 participated. Sources of referral to this study included support groups for HCV-infected patients (28%), physician offices (17%), family/friend referrals (12%), online postings (13%), and other entities (31%), such as drug treatment centers and shelters.

To qualify for study inclusion, patients must have reported a diagnosis of HCV infection as well as a positive diagnostic test. Recruiting criteria did not specify whether the HCV infection was to be acute or chronic. Currently treated and previously treated individuals were required to answer additional questions about their treatment (e.g., treatment type and duration) to qualify for the study. Previously treated patients must have received a course of treatment within the previous five years.

Potential participants were excluded if they had received treatment with a DAA. Other exclusion criteria included a diagnosis of HIV/AIDS, schizophrenia, or major contraindications for treatment (e.g., individuals undergoing cancer treatment or with decompensated cirrhosis, liver failure, liver cancer, or severe cardiac disease). People employed by a healthcare product manufacturer were excluded, as were individuals who had received payment for market research in the prior 30 days.

Pre-recruitment targets were specified with respect to age groups, gender, race, and treatment experience in an attempt to ensure adequate representation of the HCV-infected population in the United States. Table 1 provides a summary of the pre-recruitment targets for the study population.

**Table 1:** Pre-recruitment study sample targets

**Patient characteristic**	**Target/soft quota**
Gender (male),%	≥ 60
Age (median), years	50
Race/ethnicity,%	
Hispanic/Latino	10
American Indian/Alaskan Native	1
Asian/Native Hawaiian/Other Pacific	2
Black/African American	23
Caucasian/White	63
Other	1

### Study design and procedures

A systematic literature review identified key relevant studies and informed the development of a standardized interview guide. Between May 2011 and August 2011, two trained interviewers conducted 60-minute one-on-one telephone interviews consisting of open‒ and closed-ended questions about HCV infection and treatment initiation, maintenance, and completion. Interviewers selected one of five differently ordered sets of closed-ended question (i.e., same questions, different order) to reduce question order bias; interviewers ensured an equal distribution of the five ordered sets. For the 33 closed‒ended questions about factors that might influence treatment decisions, participants assigned a rating from 1 (not at all important) to 5 (extremely important) and provided verbal explanation of why they thought each factor was encouraging or discouraging in initiating and completing HCV treatment (Table 2).

**Table 2:** List of factors potentially affecting HCV treatment decisions*

	
1	Possible future health problems you expect from not treating Hepatitis C
2	Expected effectiveness of treatment (in terms of the treatment’s typical impact or lack of impact on virus levels)
3	Expected overall side effects of treatment
4	Expected depression side effects in particular
5	Expected flu-like side effects in particular
6	Expected fatigue side effects in particular
7	Amount of time needed to finish the entire treatment
8	The need to inject one of the treatment medications with a needle
9	Having to remember to take several medications according to a schedule from the doctor
10	Possible Hepatitis C treatment alternatives not discussed by your doctor
11	Need for a liver biopsy^a^
12	How Hepatitis C, the disease, has affected (or not affected) other people’s lives
13	How Hepatitis C treatment has affected other people’s lives
14	The stage of your Hepatitis C (for example, your liver status)
15	Whether or not you had Hepatitis C symptoms
16	Other health issues in addition to Hepatitis C, *[for women]* including pregnancy or possible pregnancy
17	Any substance abuse issues (alcohol or recreational street drugs)
18	The need for more information about Hepatitis C treatments
19	The extent of your will power when you decide to do something (such as starting a treatment)^a^/ The extent of your will power when you decide to do something (such as finishing a treatment)^b^
20	The effect of the condition of Hepatitis C on your ability to reach life goals
21	The effect of the condition of Hepatitis C on the lives of others, such as your family members
22	The effect treatment might have on your ability to meet work responsibilities^a^*/* The effect of the treatment on your ability to meet your work responsibilities^b^
23	The effect treatment might have on your ability to meet family responsibilities^a^*/* The effect of the treatment on your ability to meet your family responsibilities^b^
24	Your ability to pay for treatment
25	The effect treatment might have on your ability to earn money^a^/ The effect of treatment on your ability to earn money^b^
26	The stability of your housing situation
27	The emotional support you could expect from friends, family, support groups, and/or religion if you were to start treatment^a^/ Emotional support from your friends, family, support groups, and/or religion^b^
28	Your doctor’s advice
29	Your relationship with doctors and nurses in terms of the encouragement and knowledge they typically provide
30	Organizational help from doctors and nurses in things like managing appointments and helping with medications
31	How easy or hard it is to see doctors or nurses (for example traveling to the doctor’s office and making appointments)
32	Potential for being treated differently or judged if you were to start treatment^a^/ Being treated differently or judged because of the treatment^b^
33	Your ability to get information during treatment about your virus levels and how likely the treatment will work for you^a^/ Information during treatment about your virus levels and how likely the treatment will work for you^b^

*Study participants were asked to provide an importance rating for each factor. Naïve patients provided ratings in regards to HCV treatment initiation; unless otherwise noted, all other patients provided ratings based on the factors’ importance to treatment initiation and completion.

^a^Patients rated the factor only in regards to importance to HCV treatment initiation.

^b^Patients rated the factor only in regards to importance to HCV treatment completion.

### Data analysis

All interviews were recorded and transcribed verbatim with participant permission. Transcripts were subsequently coded by one researcher and reviewed by a second researcher to ensure the accuracy of the dataset. Coded responses were aggregated and summarized for the entire study population and by participant subgroup. For analytic purposes, factors described as encouraging by a participant were assigned a positive importance rating, factors described as discouraging were assigned a negative importance rating, and factors that were neither discouraging nor encouraging for a participant were assigned a zero value.

Descriptive statistics were employed to characterize study findings; analysis of variance (ANOVA) procedures were used to assess statistical significance in all study comparisons. Given that treatment motivators may depend on treatment history, patient groups selected for comparisons are treatment naïve patients, currently treated patients, patients who have completed treatment, and patients who have discontinued treatment. Differences between groups were considered significant at p-values ≤0.05.

## Results

### Patient demographic and clinical characteristics

Participants included 120 adults with HCV infection: 30 treatment naïve, 30 currently treated, and 60 previously treated - of whom 30 had completed treatment and 30 had discontinued treatment. Fifteen of the patients who had completed treatment were cured, while 15 had relapsed or had non-response. Fifty-six percent of the study participants were male; 63% were white/Caucasian (Table 3). Median age was 52 years (mean: 49; SD: 11.4), which was comparable to the median age of the HCV-infected population in the United States [17]. The geographic distribution of the study participants was as follows: West (24%), Northeast (21%), Midwest (18%), and South (38%). Patients who had completed treatment were significantly older than other patient subgroups, with mean age of 57 years (SD: 8.4; p < 0.001) compared with mean age of 42 years (SD: 13.2) in the treatment naïve group, 48 years (SD: 9.7) in the currently treated group, and 50 years (SD: 9.6) in the group who had discontinued treatment. In addition, duration of disease was longer for patients who had completed treatment (10.4 years; SD: 6.4; p < 0.001) than for other groups: naïve (4.2 years; SD: 3.2), current (5.4 years; SD: 5.5), and discontinued (9.1 years; SD: 5.0).

**Table 3:** Demographic and clinical characteristics of study participants by treatment* experience

**Patient characteristics**	**All (n = 120)**	**Completed (n = 30)**	**Discontinued (n = 30)**	**Current (n = 30)**	**Naïve (n = 30)**
Age in years, mean(±SD)	49.1 (±11.6)	56.8 (±8.4)	49.9 (±9.6)	47.6 (±9.7)	42.0 (±13.2)
Male, n (%)	67 (56)	17 (57)	16 (53)	17 (57)	17 (57)
Married, n (%)	29 (24)	11 (37)	8 (27)	7 (23)	3 (10)
Living alone at time of treatment, n (%^)a^	34 (38)	12 (40)	10 (33)	12 (40)	NA
***Race/ethnicity, n (%)***^***b***^					
Caucasian/White	76 (63)	25 (83)	16 (53)	22 (73)	13 (43)
African American/Black	28 (23)	3 (10)	8 (27)	6 (20)	11 (37)
Hispanic/Latino	12 (10)	2 (7)	5 (17)	1 (3)	4 (13)
Asian/Native Hawaiian/Other Pacific Islander	3 (3)	0 (0)	1 (3)	1 (3)	1 (3)
American Indian/Alaskan Native	1 (1)	0 (0)	0 (0)	1 (3)	0 (0)
Other	1 (1)	0 (0)	0 (0)	0 (0)	1 (3)
***Highest education level, n (%)***^***b***^					
Bachelors/graduate degree	28 (23)	11 (37)	8 (27)	6 (20)	3 (10)
Some college/2 year associate degree	50 (42)	11 (37)	13 (43)	13 (43)	13 (43)
High school diploma/GED	32 (27)	7 (23)	7 (23)	10 (33)	8 (27)
Less than high school	10 (8)	1 (3)	1 (7)	1 (3)	6 (20)
***Health insurance status when deciding whether to start treatment , n(%)***^***c***^					
Private	61 (51)	19 (63)	19 (63)	13 (43)	10 (33)
No health insurance	26 (22)	4 (13)	4 (13)	9 (30)	9 (30)
Medicaid only	19 (16)	3 (10)	6 (20)	5 (17)	5 (17)
Medicare or Medicaid/Medicare dual	9 (8)	2 (7)	1 (3)	1 (3)	5 (17)
Military insurance/TRICARE/VA	5 (4)	2 (7)	0 (0)	2 (7)	1 (3)
***Comorbid conditions, n (%)***^***d***^					
Depression	62 (52)	10 (33)	16 (53)	19 (63)	17 (57)
Anxiety	53 (44)	9 (30)	11 (37)	15 (50)	18 (60)
Compensated cirrhosis	18 (15)	5 (17)	8 (27)	4 (13)	1 (3)
Diabetes	9 (8)	3 (10)	4 (13)	1 (3)	1 (3)
Time since HCV diagnosis in years, mean (±SD)	7.3 (±5.7)	10.4 (±6.4)	9.1 (±5.0)	5.4 (±5.5)	4.2 (±3.2)

*Treatment refers to pegylated interferon and ribavirin (PR).

^a^Living alone at the time of treatment was not assessed for the treatment-naïve population, therefore the total denominator for all patients = 90.

^b^Percentages may not add to 100% because of rounding.

^c^Current health insurance status (i.e., status at the time of the interview) is captured for treatment-naïve patients.

^d^Patients may have more than one comorbid condition.

### Factors encouraging and discouraging patients to initiate treatment

**Figure 1:**
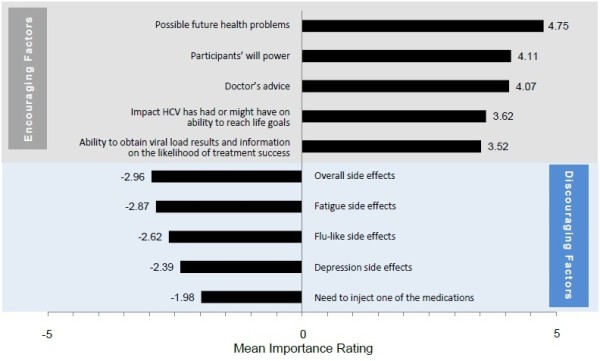
Most important factors encouraging and discouraging the initiation of HCV treatment (n = 120).

**Figure 2:**
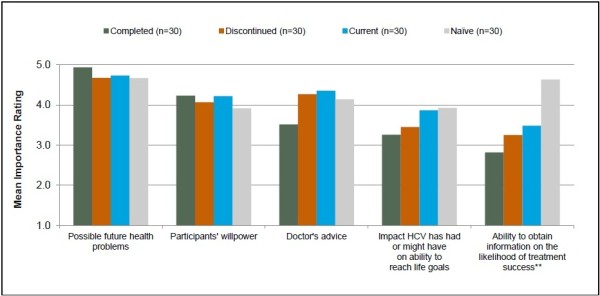
Most important factors encouraging the initiation of HCV treatment by subgroup. ** The treatment mentioned in subgroup descriptions refers to treatment with pegylated interferon and ribavirin (PR). **Difference between groups is statistically significant at p ≤ 0.05.Sample sizes may vary for individual factors because of missed questions or participant non-response.

The five factors reported as most encouraging for treatment initiation (followed by the mean importance scores) were possible future health problems from not treating HCV infection (4.8), participant’s willpower (4.1), doctor’s advice (4.1), the impact that HCV had or might have on reaching life goals (3.6), and the ability to obtain information during treatment on the likelihood of treatment success (3.5). The most discouraging factors were: the overall side effects of treatment (−3.0), fatigue (−2.9), flu-like symptoms (−2.6), depression (−2.4), and the need to inject one of the treatment medications (−2.0) (Figures 1 and 2).

#### Possible future health problems associated with HCV infection

All four patient subgroups assigned similar importance ratings and indicated similar concerns with disease progression when considering the initiation of HCV treatment (p = 0.542). Fears of progressing towards cirrhosis, liver failure, liver cancer, and death were commonly mentioned. Comments from two currently treated patients are below.

Current: *“*[Possible future health problems] *was the most important aspect in my mind at that point. I did not want to end up to be an invalid or an extremely sickly person for the rest of my life because I didn’t or wasn’t willing to try an approach to hopefully fight and heal a disease, or at least bring something that’s out of balance more in balance.”*

Current: *“I didn‘t want to die of liver cancer or cirrhosis.”*

Among naïve patients, however, concern about future health problems associated with HCV infection was often not enough of a concern to seek treatment immediately. Other issues, such as expected medication side effects (cited by 60% of naïve patients), physician recommendations (40%), and disease stage (40%), were reported to have influenced their decisions to decline treatment as well.

#### Participants’ willpower

All four study subgroups reported similar ratings for the importance of inner strength/willpower as motivation to start HCV therapy (p = 0.938).

Current: *“I mean once you commit yourself that’s it. I’m not a middle of the road guy. It’s either 100% or don’t do it at all.”*

As shown in the comment below, some patients who recognized their lack of willpower stressed the importance of finding strength from other sources.

Completed: *“My willpower has never been very good. That’s why I got support. They help you with your willpower. Willpower—it’s important. We got to have the will to do it, but the support group helps you get that will better lined up.”*

#### Doctor’s advice

Study participants generally valued the opinion of their clinicians and recognized their expertise as important for guiding them through the treatment decision process. Patients reported doctor’s advice to be important in encouraging their decision to initiate treatment; no differences were observed between subgroups (p = 0.294). Specifically, patients mentioned that they valued their clinician’s training, experience, and knowledge. Patients also reported that they were further encouraged by their clinician’s positive feedback and confidence regarding treatment effectiveness and associated outcomes. Forty percent of naïve patients indicated that they had deferred HCV treatment initiation because of their clinician’s recommendation.

Discontinued: *“I have no medical training or skills. I have to rely on what my doctor suggests or what he recommends in order to make a decision as to what I’m going to do.”*

The patients quoted below noted that their doctors had provided information that allowed them to make treatment decision themselves.

Completed: *“The doctor pretty much left it up to me. He didn‘t advise me to go for it or not to go for it. It was not a question of live or die thing because I was at such a low stage.”*

Completed: *“*[The doctors] *left it* [the treatment decision] *up to me. They didn‘t force it on me. It was if I made the decision I would probably be more likely to go through it, than if they made the decision.”*

Despite overall clinician praise, the comments below show some disappointment in clinicians’ advice regarding treatment.

Completed: *“I sometimes wish that he were a little more aggressive, a little less conservative in treating this virus.”*

Current: *“I am really not sure how much he knew about this disease.”*

Current: *“He didn't seem to care. Like I said, in the beginning all he said was, ‘We can give you a shot.’”*

#### Impact of HCV infection on ability to reach life goals

Across all patient subgroups, the impact of HCV infection on patients’ ability to reach life goals was important to the treatment decision. Goals reported as unattainable with HCV infection included living a happy, healthy, and long life, pursuing and advancing in a career, furthering education, participating in recreational activities (e.g., travel and sports), and starting a family or spending time with existing family. Patients who had previously completed treatment, assigned slightly lower importance to this factor; differences in the mean importance rating were not statistically significant between subgroups (p = 0.638).

Naïve: *“I want to be there for my kids, live to see them grow up. I have a relative who is dying of hepatitis C.”*

Naïve: *“If I get sick because I have hep C and I can’t physically work, it’s going to affect all kinds of things. It’s going to affect my income. It’s going to affect my job. It’s going to affect my home life.”*

#### Ability to get information on the likelihood of treatment success

Sixty-nine percent of all participants (77% [naïve ]; 67% [currently or previously treated]) indicated that the ability to know the likelihood of treatment success would encourage them to initiate treatment. The naïve group assigned a greater importance rating to the ability to obtain this information compared with all other participant groups (p = 0.005).

**Figure 3:**
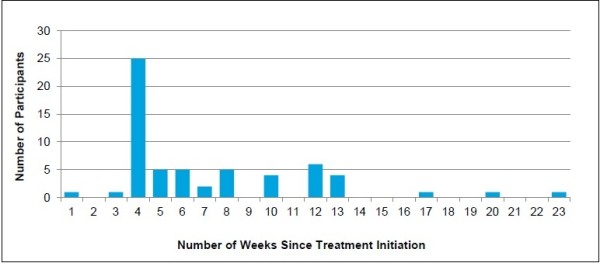
Preferred timing for data about likelihood of treatment success for participants motivated by this factor (n = 61*). *An additional 13 patients mentioned “as soon as possible” and four mentioned “early on during treatment.”

Of the 69% of participants who reported that this information would encourage them to start treatment, 29% indicated that they would like to know (or would have liked to have known) these results within four weeks of treatment initiation. An additional 16% of participants motivated by this information indicated that they would prefer this information “as soon as possible.” The median preferred timing for viral load results was reported to be five weeks across all study participants reporting to be motivated by this type of information (Figure 3). For naïve participants, the reported timing preferred for viral load results was later (median: 10 weeks).

Study participants were generally interested in understanding the effectiveness of therapy during the course of treatment. Some patients specifically indicated that having the opportunity to review these results afforded them some level of control over their treatment.

Current: *“I want to make sure that if I’m going to undergo something, it’s going to work. I just don’t want to waste my time with something.”*

Discontinued: *“Just knowing what’s going on, for me, is very, very important. I don’t like to be in the dark, and I like to be able to make decisions or just know what’s going on.”*

#### Side effects of HCV treatment

The four most important factors that discouraged treatment initiation among study participants were related to overall and individual treatment side effects such as fatigue, flu-like symptoms, and depression. Forty percent of all study participants anticipated fatigue, 40% anticipated flu-like symptoms, and 25% anticipated depression; however, the potential for other side effects was of concern.

Naïve: *“I hear horrible stories on the internet.”*

Naïve: *“I had seen somebody on it and they were very sick and very weak and tired. Then if you do something with it and you drink or something like that, you know, you could get even worse. Then you like start throwing up and get all depressed and all that, so I’m scared to take it.”*

Current: *“My thoughts were am I going to be able to keep up with all of this. Am I going to have the energy? Am I going to be well enough to keep doing what I'm doing?”*

The importance assigned to side effects was higher for the naïve and current subgroups than for the previously treated subgroup (p = 0.004). In the naïve participant group, 60% of participants indicated that treatment-related side effects were a primary reason for not initiating treatment.

#### Need to inject one of the treatment medications

The method of treatment administration also factored into patients’ treatment decisions, with similar importance ratings across the four subgroups (p = 0.507). Fear of needles, discomfort with self-injections, and reminders of illicit drug use were cited as issues that discouraged treatment initiation, as seen in the quotations below.

Completed: *“I don't like shots and I certainly didn’t want to have to give myself one.”*

Naïve: *“I don't like needles because of my IV drug history. It's a trigger for me.”*

Previously treated patients offered some suggestions for facilitating treatment injections. Eleven percent of previously treated participants specifically highlighted the importance of training materials and guidance from their clinicians with respect to injection sites, general technique, and numbing or distraction strategies. Ten percent of previously treated patients suggested having someone else administer the medications.

### Factors encouraging and discouraging patients to complete treatment

**Figure 4:**
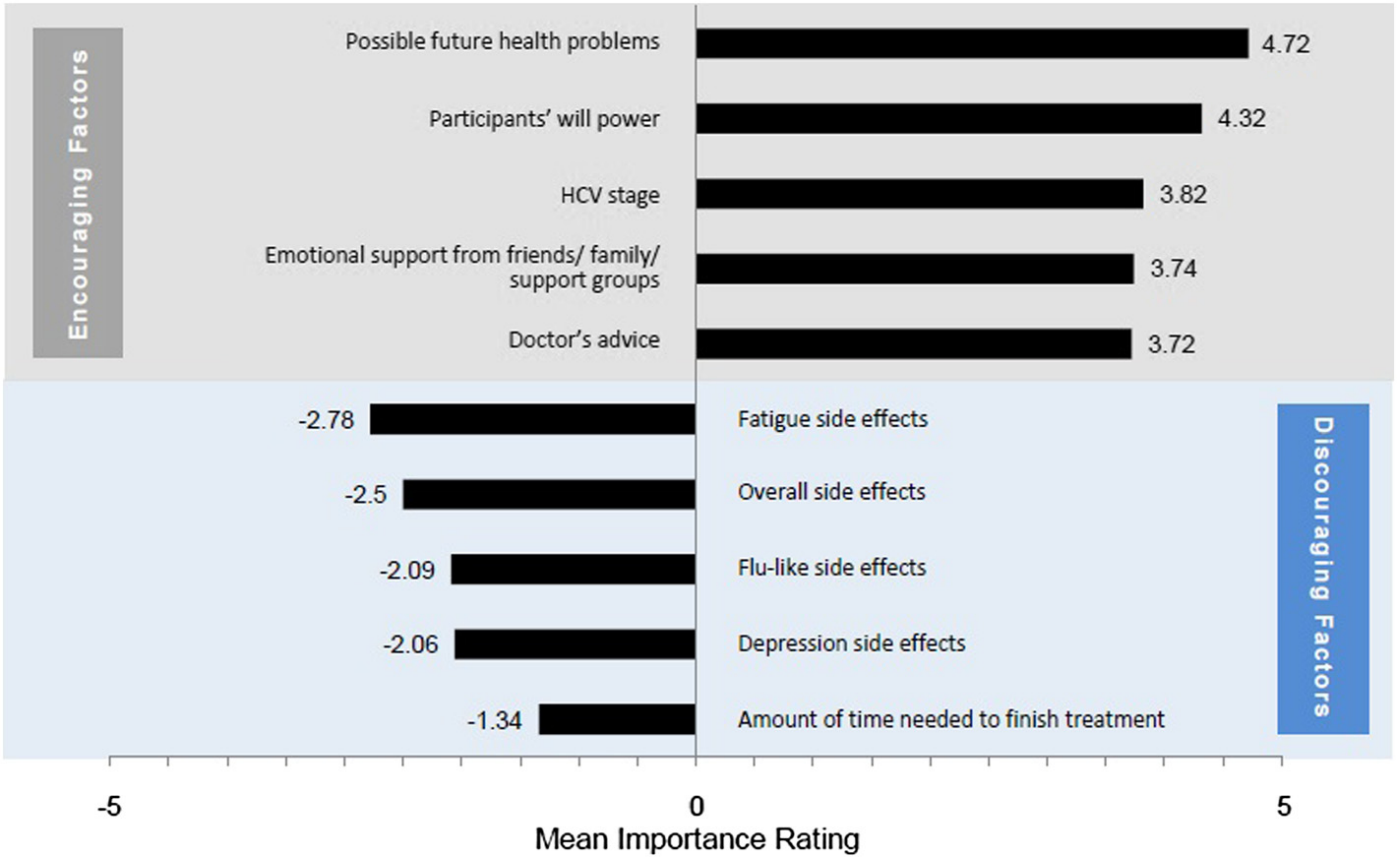
Most important factors encouraging and discouraging HCV treatment completion.

**Figure 5:**
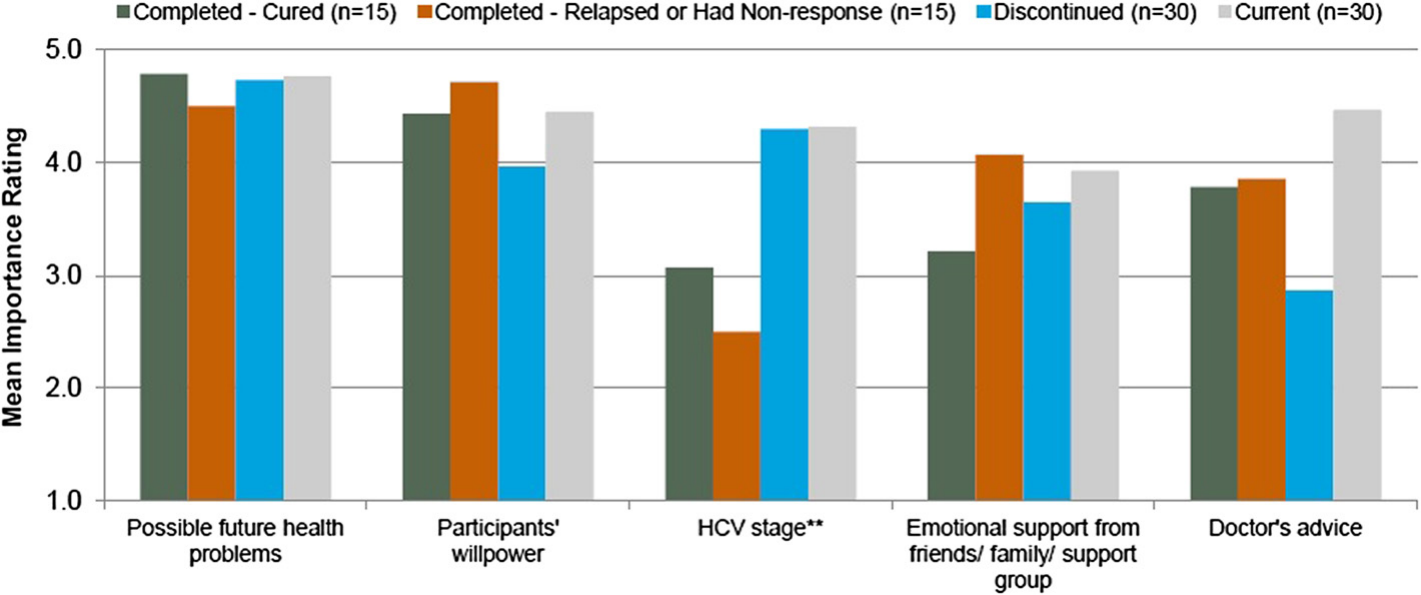
Most important factors encouraging HCV treatment completion by subgroup. ** The treatment mentioned in subgroup descriptions refers to treatment with pegylated interferon and ribavirin (PR). **Difference between groups is statistically significant at p ≤ 0.05.Sample sizes may vary for individual factors because of missed questions or participant non-response.

The top five factors reported as encouraging for the adherence to and completion of treatment were the following (Figures 4 and 5): possible future health problems from not treating HCV infection, patients’ willpower, stage of disease, available emotional support, and doctor’s advice. Although the stage of HCV disease and the availability of emotional support from others (e.g., friends, family, support groups) were not in the top five factors encouraging treatment initiation, they became more important in decisions to complete treatment. We provide more detail on these two factors in the sections below; we do not provide further detail on the other three factors because patients’ comments were almost identical to the comments provided in the section on treatment initiation.

The two most important factors discouraging HCV treatment adherence or completion were treatment-related side effects and overall treatment duration.

#### Stage of HCV disease

In particular, study participants who were currently treated or had discontinued treatment reported that the stage of their liver disease was an important factor for adhering to the prescribed HCV regimen and for completing treatment. As their disease had progressed, so did their desire to complete HCV therapy.

Completed: *“I was really wanting to get rid of it, so it wouldn‘t do any worse damage.”*

#### Availability of support

Previously treated patients (i.e., patients who had finished or discontinued treatment) and currently treated patients reported that support from family, peers, and health care providers encouraged treatment adherence and completion, as did religious faith.

Current: *“I think definitely anybody going into it should go to a support group and talk to people that have been through it. That’ll definitely help you. It really raises your hopes when you see people that it has worked with, and they can tell you about their experience, what they went through with it, and how their lives are changed after going through it.”*

Discontinued: *“Just moral support from friends and family and reading online forums of people's suggestions, like doing the injection at night time so you sleep through the initial worse side effects. Just try to be informed and take other peoples’ suggestions to heart.”*

#### Side effects of HCV treatment

On a scale of 1 (not at all bothersome) and 5 (extremely bothersome), the mean rating of treatment side effects for the previously and currently treated participants combined was −3.69 (−3.38 [current], -3.90 [discontinued], and −3.78[completed], respectively) (These differences were not significant.) Side effects were more bothersome for patients who had discontinued treatment than for other subgroups, and 43% of patients who had discontinued mentioned side effects as a reason for stopping HCV treatment.

Survey participants offered several suggestions for strategies to overcome treatment side effects. The use of additional medications (e.g., anti-nausea drugs, antidepressants, over-the-counter flu medications) was reported as helpful, as were exercise, rest, positive thinking, and distraction. In addition, an important strategy was to take medications as part of a daily routine (i.e., before bed and injections over weekends) to avoid interference with patients’ usual activities and a work day.

Current: *“Trying to ignore them—like focus on something else—actually, do something—not just lay around feeling miserable.”*

Current: *“I do a meditation class with my acupuncturist, and acupuncture has helped through the treatment. It helped a lot for my anxiety and my moods, my nausea, and so many of the side effects.”*

Discontinued: “*Sometimes I’d just wait until Saturday—a better day, the weekend. Who cares? I don’t have to work. Do them then and then it didn’t affect me so much—the side effects.”*

#### Treatment duration

Doubts about being able to complete treatment were reported to have emerged during treatment; such doubts were tied both to the administration of medication and the medications’ side effects. As seen in the comment below, participants indicated that they would have been more willing to endure the side effects if they had known that treatment could be shorter.

Discontinued: *“If I were to have to go 12 months with these types of feelings I wouldn‘t be able to do it. But on the other hand if I had to go two more months I would have probably stayed with it.”*

## Discussion

Study findings suggest that future health problems related to chronic HCV infection and the expected efficacy and safety profile of HCV therapies are likely to impact patients’ decisions on HCV treatment initiation and completion. Such risk-benefit considerations have been reported to be more likely to increase patient satisfaction than other decision paradigms [8] and to provide a promising foundation for those seeking to increase HCV treatment adherence and completion [18]*.*

The expectation of health problems from not treating HCV infection was identified as the most important factor for encouraging therapy initiation, consistent with data from an Australian study [10]. Patients want to be in good health now and in the future but while HCV treatment may provide future health benefits, side effects make patients feel poorly in the present. Participants in our research indicated that side effects of treatment were most important both in discouraging treatment start and in challenging treatment adherence.

Early knowledge of the likelihood of treatment success was suggested to be of great value to many patients. Based on the study findings, the ability to obtain laboratory testing results in the early treatment phase could be a motivating factor for patients to seek HCV treatment and to adhere to therapy following their decision to initiate therapy. Our study findings could be relevant to those patients initiating the newer therapies available including DAA-based regimens. In such instances, guidelines issued by the American Association for the Study of Liver Diseases (AASLD) for the treatment of genotype 1 chronic HCV infection [6] should be consulted in determining the value of early on-treatment viral load assessments.

Analyses of the factors that are important to patient treatment decisions (e.g., expectation of future disease complications and treatment side effects), coupled with earlier research indicating that physicians may not understand how patients value the risks and benefits of antiviral therapy [19], suggest the utility of informing clinicians of the patient perspective regarding HCV treatment. In our research, patient suggestions on how to overcome barriers to starting HCV treatment included keeping communication open between the patient and doctors, nurses, and pharmacists. Communication and education are especially helpful given the importance of physician recommendations in patient decisions about whether or not to begin HCV therapy [8].

It is also important for clinicians to understand nuances in patients’ decision-making processes for those with and without prior HCV treatment experience. For example, side effects were more discouraging in treatment-naïve than in treatment-experienced patients, whereas the ability to get information on the chance of treatment success was identified as a higher motivating factor in treatment-naïve patients. Fear of potential side effects may subside somewhat once patients have experience with the treatment regimen whereas the ability to learn about the likelihood of treatment success early during treatment may resonate highly in treatment-naïve patients.

Our results suggest the need for clinicians to educate patients in the areas of disease progression, the potential of long-term clinical consequences if treatment is postponed, and the challenges of treatment-related side effects. Based on our research, discussion topics should also include education about treatment duration, as well as the importance of committing to treatment in light of the consequences of early treatment discontinuation. Clinician support would also include training in medication administration and management of side effects. Other research has shown that similar education programs in HCV-infected patients eased their fears and increased rates of treatment eligibility [20].

This study, along with others [21-23], highlights the importance of emotional support (family, friends, and support groups), peer-to-peer conversations, and support groups for HCV-infected patients. Many patients have reported to find support groups more useful than providers at providing health-related information [22]. In our research, patients repeatedly suggested the value of identifying individuals in comparable circumstances as a way to encourage treatment initiation. Peer education and support groups can help patients understand how others have successfully managed their disease and treatment; emotional support may also be provided by bringing together patients at similar stages of treatment.

Several limitations should be considered when evaluating study findings. First, with the exception of the rating exercises, the research methodology was qualitative. Such methods, however, provide an opportunity to gain a deeper understanding of the individual decision-making process in regards to HCV treatment initiation and completion. Second, although the importance ratings of some factors are significantly different across patient groups, the sample sizes of the patient groups may have been too small to detect statistically significant differences for other important factors. Third, true motivators may be different than reported motivators, especially for previously treated patients tasked with recalling the factors that had motivated them in the past. This is an inherent limitation of this study design. Fourth, this study employed a convenience sample, which has the potential to impact the generalizability of the results. To help counteract this potential limitation, we employed quotas for key patient characteristics reflecting the distribution of such factors in the HCV-infected population in the United States. Lastly, because the design of this study did not allow for access to medical records, we were unable to verify conclusively that all survey participants had a diagnosis of HCV infection; however, patients who reported being currently or previously treated had to answer additional questions about treatment type and duration to qualify for study inclusion.

## Conclusion

HCV-infected individuals often choose to defer initiating treatment or to discontinue therapy. Understanding potential motivators and treatment challenges from the patient perspective is an important means to identify opportunities for education and interventions that encourage initiation and completion of HCV treatment.

## Abbreviations

DAA: Direct-acting antiviral agents; HCV: Hepatitis C virus; PR: Pegylated interferon/ribavirin.

## Competing interests

LF, JA, and TG are employed by Boston Healthcare Associates, which received funding from Vertex Pharmaceuticals Incorporated for this research. At the time this research was performed, CD was employed by Boston Healthcare Associates. MV and SB are employees and stockholders of Vertex Pharmaceuticals Incorporated and may own stock or options at the company. MD is a former employee of Vertex Pharmaceuticals Incorporated and may own or may have owned stock or options in that Company at the time this research was performed.

## Authors’ contributions

LF was involved in study design and data analysis, as well as manuscript drafting and revision. JA was involved in study design, data analysis and synthesis, manuscript development, and critical revisions to the paper. CD was involved in data analysis. MV was involved in the development of the survey instrument and providing a critical review of the paper. SB provided background HCV patient market research information that informed the design of the survey. MD participated in results interpretation and provided critical revisions and review of the manuscript. TG was responsible for study conceptualization and design and analysis, as well as critical revisions of the paper. All authors read and approved the final manuscript.

## Pre-publication history

The pre-publication history for this paper can be accessed here:

http://www.biomedcentral.com/1471-2334/13/234/prepub

## References

[B1] Chack E Talal AH Sherman KE Schiff ER Saab S Hepatitis C virus infection in USA: an estimate of true prevalence Liver Int 2011 31 8 1090 1101 10.1111/j.1478-3231.2011.02494.x 21745274

[B2] Centers for Disease Control and Prevention Hepatitis C FAQs for the public 2009 [http://www.cdc.gov/hepatitis/c/cfaq.htm]

[B3] Centers for Disease Control and Prevention Hepatitis C general fact sheet 2010 http://www.cdc.gov/hepatitis/HCV/PDFs/HepCGeneralFactSheet-BW.pdf

[B4] National Research Council Hepatitis and liver cancer: a national strategy for prevention and control of hepatitis B and C 2010 Washington, DC: The National Academies Press 25032367

[B5] Ghany MG Nelson DR Strader DB Thomas DL Seeff LB An update on treatment of genotype 1 chronic hepatitis C virus infection: 2011 practice guideline by the American Association for the Study of Liver Diseases Hepatology 2011 54 4 1433 1444 10.1002/hep.24641 21898493 PMC3229841

[B6] Ghany MG Strader DB Thomas DL Seeff LB Diagnosis, management, and treatment of hepatitis C: an update Hepatology 2009 49 4 1335 1374 10.1002/hep.22759 19330875 PMC7477893

[B7] Bacon BR Mchutchison JG Treatment issues with chronic hepatitis C: special populations and pharmacy strategies Am J Manag Care 2005 11 Suppl 10 S296 S306 16232013

[B8] Fraenkel L Mcgraw S Wongcharatrawee S Garcia-tsao G What do patients consider when making decisions about treatment for hepatitis C? Am J Med 2005 118 12 1387 1391 10.1016/j.amjmed.2005.05.029 16378783

[B9] Strathdee SA Latka M Campbell J Factors associated with interest in initiating treatment for hepatitis C Virus (HCV) infection among young HCV-infected injection drug users Clin Infect Dis 2005 40 Suppl 5 S304 S312 15768339 10.1086/427445 PMC2196220

[B10] Mcnally S Temple-smith M Sievert W Pitts MK Now, later or never? Challenges associated with hepatitis C treatment Aust N Z J Public Health 2006 30 5 422 427 10.1111/j.1467-842X.2006.tb00457.x 17073222

[B11] Khokhar OS Lewis JH Reasons why patients infected with chronic hepatitis C virus choose to defer treatment: do they alter their decision with time? Dig Dis Sci 2007 52 5 1168 1176 10.1007/s10620-006-9579-1 17357838

[B12] Treloar C Holt M Drug treatment clients‘ readiness for hepatitis C treatment: implications for expanding treatment services in drug and alcohol settings Aust Health Rev 2008 32 3 570 576 10.1071/AH080570 18666886

[B13] Grebely J Genoway KA Raffa JD Barriers associated with the treatment of hepatitis C virus infection among illicit drug users Drug Alcohol Depend 2008 93 1–2 141 147 17997050 10.1016/j.drugalcdep.2007.09.008

[B14] Bernstein D Kleinman L Barker CM Revicki DA Green J Relationship of health-related quality of life to treatment adherence and sustained response in chronic hepatitis C patients Hepatology 2002 35 3 704 708 10.1053/jhep.2002.31311 11870387

[B15] Mchutchison JG Manns M Patel K Adherence to combination therapy enhances sustained response in genotype-1-infected patients with chronic hepatitis C Gastroenterology 2002 123 4 1061 1069 10.1053/gast.2002.35950 12360468

[B16] Lore V Amorosa VK Localio AR Adherence to hepatitis C virus therapy and early virologic outcomes Clin Infect Dis 2009 48 2 186 193 10.1086/595685 19086908 PMC2668718

[B17] Centers for Disease Control and Prevention Viral Hepatitis Surveillance – United States 2010 [http://www.cdc.gov/hepatitis/Statistics/2010Surveillance/index.htm]23719564

[B18] Jenner A Scott A Circulating beliefs, resilient metaphors and faith in biomedicine: hepatitis C patients and interferon combination therapy Sociol Health Illn 2008 30 2 197 216 10.1111/j.1467-9566.2007.01061.x 18290932

[B19] Cotler SJ Patil R Mcnutt RA Patients‘ values for health states associated with hepatitis C and physicians’ estimates of those values Am J Gastroenterol 2001 96 9 2730 2736 10.1111/j.1572-0241.2001.04132.x 11569703

[B20] Evon DM Simpson K Kixmiller S A randomized controlled trial of an integrated care intervention to increase eligibility for chronic hepatitis C treatment Am J Gastroenterol 2011 106 10 1777 1786 10.1038/ajg.2011.219 21769136 PMC3683982

[B21] Munoz-plaza CE Strauss S Astone-twerell J Exploring drug users’ attitudes and decisions regarding hepatitis C (HCV) treatment in the U.S Int J Drug Policy 2008 19 1 71 78 10.1016/j.drugpo.2007.02.003 18312822 PMC2698452

[B22] Jessop AB Cohen C Burke MM Conti M Black M Hepatitis support groups: meeting the information and support needs of hepatitis patients Gastroenterol Nur 2004 27 4 163 169 10.1097/00001610-200407000-00004 15326401

[B23] Sylvestre DL Zweben JE Integrating HCV services for drug users: a model to improve engagement and outcomes Int J Drug Policy 2007 18 5 406 410 10.1016/j.drugpo.2007.01.010 17854729

